# Simultaneous Determination of Pyrethrins, Pyrethroids, and Piperonyl Butoxide in Animal Feeds by Liquid Chromatography-Tandem Mass Spectrometry

**DOI:** 10.3390/toxins15060401

**Published:** 2023-06-17

**Authors:** Xin Xu, Lisa A. Murphy

**Affiliations:** Pennsylvania Animal Diagnostic Laboratory System Toxicology Laboratory, New Bolton Center, University of Pennsylvania School of Veterinary Medicine, 382 W Street Rd., Kennett Square, PA 19348, USA

**Keywords:** feed contamination, pyrethrin, pyrethroid, piperonyl butoxide, livestock feed, poultry feed, liquid chromatography–tandem mass spectrometry

## Abstract

The presence of insecticides like pyrethrins and synthetic pyrethroids, combined with the synergist piperonyl butoxide, in animal feeds can pose a risk to both animal and human health by contaminating the food chain. In this study, a simple and fast method was developed for the simultaneous determination of these compounds in contaminated animal feeds using liquid chromatography–tandem mass spectrometry (LC-MS/MS). Sample preparation was carried out using a QuEChERS-based approach, and the method was validated with acceptable accuracy ranging from 84 to 115% and precision below 10%. The limit of detection (LOD) and limit of quantification (LOQ) were between 0.15 and 3 and 1 and 10 µg/kg, respectively. The method detected insecticide contaminations in various livestock and poultry feeds. Furthermore, the method was applied to a toxicology case, where it identified and quantified piperonyl butoxide and deltamethrin in the submitted horse feed sample. These results demonstrate that the method can be a valuable tool in animal health and food safety diagnostic applications, as well as veterinary toxicology investigations concerning pyrethrin-related feed contamination.

## 1. Introduction

Pyrethrins and pyrethroids are widely used insecticides in agriculture and veterinary medicine to control various pests [1,2,3]. Pyrethrins are naturally occurring insecticides derived from the flowers of chrysanthemum plants. These natural compounds have relatively low stability when exposed to various environmental factors such as heat and light [4]. This inherent instability has prompted the development of pyrethroids, synthetic analogs of pyrethrins. Pyrethroids were designed to mimic the structure and insecticidal activity of pyrethrins, while possessing enhanced stability, allowing them to persist in the environment for extended periods and withstand degradation caused by environmental factors. Both pyrethrins and pyrethroids are highly valued for their efficacy in protecting livestock and domestic animals from insects such as flies, mosquitoes, ticks, mites, and lice [1,2,3]. They share a similar mechanism of action by targeting the nervous system of insects, primarily affecting the voltage-gated sodium channels [5]. These compounds bind to the sodium channels and cause prolonged opening or delayed closure, which leads to hyperexcitability and paralysis of the insects. Additionally, pyrethrins and pyrethroids can have effects on the voltage-dependent chloride channels in insects [4], which may further increase nerve cell excitability and contribute to insecticidal effects.

Piperonyl butoxide (PBO) is a chemical synergist commonly employed in conjunction with pyrethrins and pyrethroids for insect control [1]. PBO acts by inhibiting the activity of cytochrome P450 enzymes present in insects [1,6]. These enzymes play a vital role in the breakdown and detoxification of insecticides. By inhibiting these enzymes, PBO prevents insects from efficiently metabolizing and eliminating the pyrethrins and pyrethroids [4]. As a result, the insecticides remain active for extended periods, increasing their potency and effectiveness against pests.

When livestock and other domestic animals ingest feed or water contaminated with pyrethrins and pyrethroids or are directly exposed to these insecticides through the application, the chemicals can potentially enter the food chain through animal-derived products [2]. As a result, people can be exposed to these insecticides by consuming contaminated animal products [7]. Although pyrethrins and pyrethroids are generally considered to have low toxicity to mammals, research suggests they can still cause adverse health effects in animals and humans. Exposure to these chemicals has been linked to neurotoxicity, reproductive toxicity, developmental disorders, immunotoxicity, and carcinogenicity [2,8,9,10]. Animal studies have demonstrated that pyrethrins and pyrethroids can impact the nervous system in mammals. These compounds have been found to interfere with neurotransmission, leading to a variety of neurological effects such as hyperexcitability, tremors, and seizures [1,4]. In addition, these chemicals may possess endocrine-disrupting properties, affecting both the female reproductive system and male fertility, and potentially leading to reproductive and developmental disorders [2,10]. Exposure to pyrethrins and pyrethroids has also been shown to alter immune responses and have detrimental effects on the immune system [2]. Moreover, studies have shown that exposure to pyrethroids can increase the risk of developing cancers [2,10]. Furthermore, the presence of PBO could potentially enhance the potency of pyrethrins and pyrethroids and lead to an elevated risk of adverse health effects in animals and humans [1,4].

Gas chromatography- (GC) and liquid chromatography (LC)-based techniques have been widely used to analyze pyrethrins, pyrethroids, and PBO in animal tissues and foods of animal origin [11,12,13,14,15,16,17,18,19,20]. GC coupled with electron capture detector or mass spectrometry (MS) detection has been employed to detect pyrethroids in fish tissues, livestock meat, milk, and eggs [12,13,14,15,16,17]. LC coupled with ultraviolet detector or MS detection has also been utilized to determine pyrethroids and PBO in animal tissues, fat, milk, and eggs [18,19,20]. However, all these studies have only investigated contamination in the final animal products. One recent study reported the occurrence of PBO in diets of dairy cattle using an LC-MS-based method [21]. This study focused on one type of animal feed and did not include pyrethroids or pyrethrins in its analysis. Therefore, there is a lack of research on the simultaneous determination of pyrethrins, pyrethroids, and PBO in various animal feeds, which could be crucial in determining the primary source of pyrethrins-related contaminations in cases of animal poisoning or in animal-derived foods. The objective of this study was to establish a simple, efficient, and fast method for simultaneous quantification of pyrethrins, pyrethroids, and PBO in livestock and poultry feeds using liquid chromatography–tandem mass spectrometry (LC-MS/MS). The pyrethrins included in the analysis were pyrethrin I, pyrethrin II, cinerin I, cinerin II, jasmolin I, and jasmolin II. The three primary pyrethroids included in the study were cypermethrin, deltamethrin, and permethrin [1,3,10].

## 2. Results and Discussion

### 2.1. LC-MS/MS Conditions

MS ionization modes were first investigated for each compound. It was observed that pyrethrins, pyrethroids, and PBO had more abundant precursor ions when analyzed in the positive electrospray ionization mode. Pyrethrins were found to form precursor ions as [M + H]^+^ adducts, while pyrethroids and PBO formed precursor ions as [M + NH_4_]^+^ adducts. These results were consistent with the ionization patterns of these compounds reported in previous LC-MS/MS studies [19,20].

Different LC mobile phase additives were further investigated to assess the compounds’ ionization efficiency and chromatographic separation. The following mobile phases’ additives in water and methanol were evaluated: 0.1% formic acid, 5 mM ammonium acetate, and a mixture of 0.1% formic acid and 5 mM ammonium acetate. It was found that the presence of formic acid reduced the peak intensities of pyrethroids and PBO, which have precursor ions in the form of [M + NH_4_]^+^ adducts. Therefore, the developed method employed mobile phases of (A) 5 mM ammonium acetate in water and (B) 5 mM ammonium acetate in methanol to analyze the target compounds.

**Figure 1:**
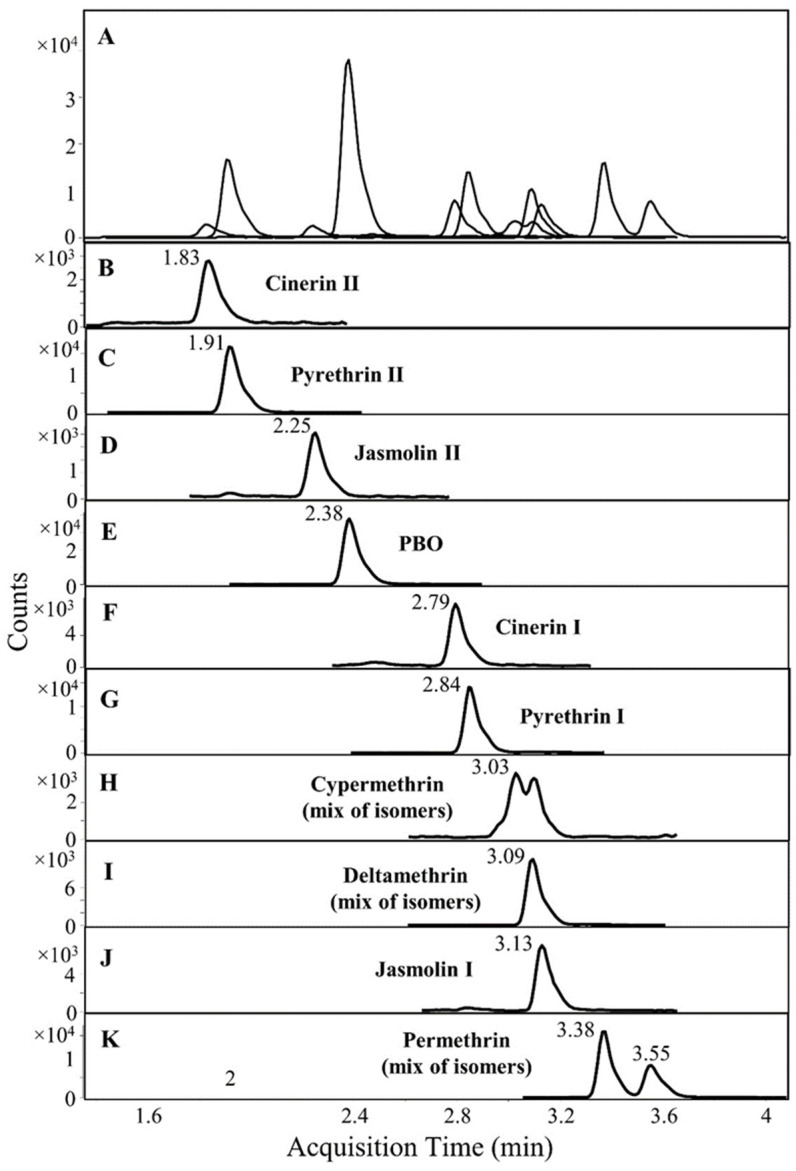
(**A**) LC-MS/MS chromatogram of eluting pyrethrins, pyrethroids, and PBO in a spiked feed sample containing 112.2 µg/kg of pyrethrin I, 55.6 µg/kg of pyrethrin II, 11.4 µg/kg of cinerin I, 7.6 µg/kg of cinerin II, 8 µg/kg of jasmolin I, 5.4 µg/kg of jasmolin I II, 200 µg/kg of cypermetherin, deltamethrin and permethrin, and 20 µg/kg of PBO. (**B**–**K**) Individual MRM chromatograms of pyrethrins, pyrethroids, and PBO.

Figure 1 shows the LC-MS/MS chromatogram of pyrethrins, pyrethroids, and PBO in a spiked feed sample. The total run time for analyzing a single sample is less than 7 min, demonstrating the method’s ability to provide rapid analysis of these contaminating chemicals in feeds. It should be noted that cypermethrin and permethrin eluted as two peaks due to their isomeric nature. The quantification of these compounds was performed by summing the peak areas of both isomers.

### 2.2. Method Validation Results

**Figure 2:**
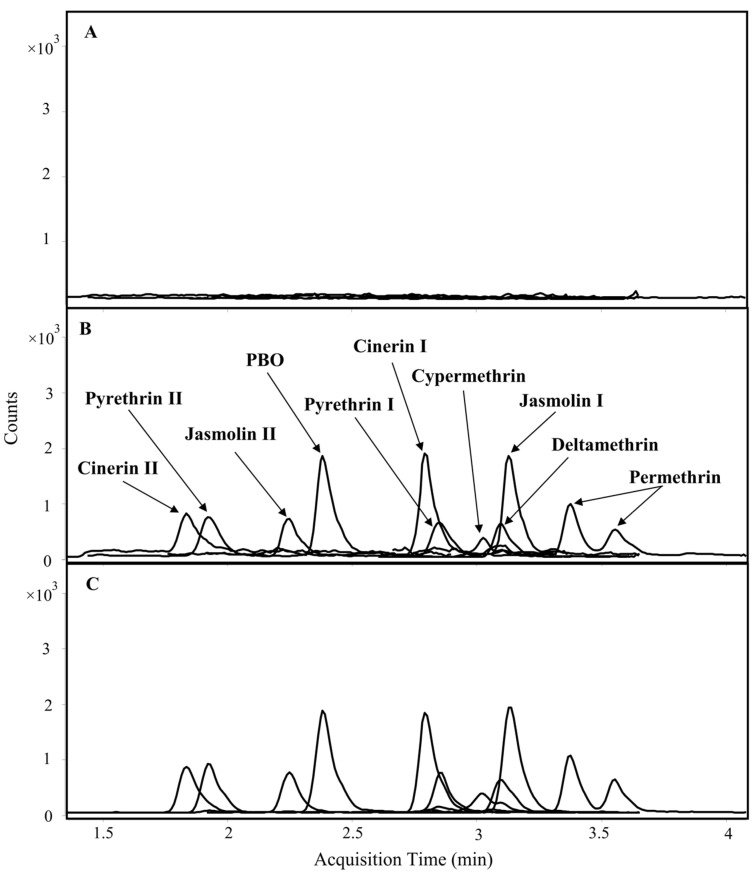
LC-MS/MS chromatograms of (**A**) blank feed sample, (**B**) blank feed spiked at the LOQ level, and (**C**) standard solution at the LOQ level.

Figure 2 presents the LC-MS/MS chromatograms of a blank feed sample, a feed sample spiked at the limit of quantification (LOQ) level, and a standard solution at the LOQ level. No interfering peaks were observed at the retention times of the analytes in the blank feed sample, confirming that the blank feed was free from endogenous interferences for the analysis of the target compounds. Table 1 presents the quantitation range of each compound in the feeds, and their corresponding limit of detection (LOD) and LOQ. All compounds showed good linearity with a coefficient of determination (R^2^) greater than 0.99. The LOD and LOQ values ranged from 0.15 to 3 and 1 to 10 µg/kg, respectively, which are similar to or better than the detection limits reported in previous studies that analyzed these compounds in animal-derived foods and feed using GC/MS or LC-MS/MS [12,14,16,17,19,20,21]. Furthermore, our present method shows additional advantages when compared to the approaches used in those previous studies, such as the use of a smaller sample size and shorter analysis run time, as shown in Table 2.

As presented in Table 3, the accuracy of all compounds at the three concentrations during both intra-day and inter-day evaluations ranged from 84 to 115%. Similarly, the precision of all compounds ranged from 1.2 to 9.1%. Overall, the method’s accuracy was within 80 to 120%, and its precision was less than 10%, demonstrating its reliability for analyzing these compounds in feed samples.

As shown in Table 1, most of the target compounds in this study experienced ion suppression with matrix effects ranging from 76% to 88%. PBO was the only compound that showed ion enhancement with a matrix effect larger than 100%. Using an internal standard (IS) for each compound minimized the variations due to matrix effects, as demonstrated by the acceptable accuracy and precision.

The accuracy of the processed QC samples stored at 10 °C remained within the acceptable range of 80–120% for the first three days. However, on the fourth day, the accuracy of pyrethrins exceeded 120%. These results suggest that processed samples can maintain stability for up to three days when stored at 10 °C.

The accuracy of the 1:50 (*w*/*w*) diluted QC samples was found to be within 85–116%, with a precision of less than 15%. These results indicate that the 1:50 (*w*/*w*) dilution is acceptable for use in feed samples with analyte concentrations exceeding the highest calibrator level.

### 2.3. Method Application in Real Feed Samples

The developed method was applied for the simultaneous determination of pyrethrins, pyrethroids, and PBO in a variety of livestock and poultry feeds obtained from the American Association of Feed Control Officials (AAFCO). Table 4 shows the presence and levels of these compounds in six cattle feeds, three horse feeds, two goat feeds, four sheep feeds, four pig feeds, and three poultry feeds. The results reveal that PBO was detected in all feed samples, with levels ranging from 1.17 to 894.8 μg/kg. Significant variations were observed both between and within different types of animal feed. The highest concentration of PBO was found in goat feed sample 1 with a value near 900 μg/kg. Deltamethrin was present in all animal feed categories except for pig feed, with high levels found in goat feed sample 1 and sheep feed sample 1 at 89.6 and 55 μg/kg, respectively. Cypermethrin was only detected in pig and poultry feed types, with high concentrations found in pig feed sample 1 (77.2 µg/kg) and poultry feed sample 2 (93.5 µg/kg). On the other hand, pyrethrins and permethrin were not detected in any of the animal feed samples analyzed. The differences in concentrations and profiles of the insecticides between and within animal feed types may be attributed to variations in pesticide stability and usage patterns during the cultivation, processing, and storage of feed ingredients.

The United States Environmental Protection Agency (EPA) has established tolerance levels for PBO and pyrethroids in various foods of animal origin, including meat, fat, and milk [22]. However, its regulations on animal feeds have a limited scope. The EPA has established a tolerance of 10 ppm for PBO residues in or on feed “when present as the result of migration” from storage bags [22]. For cypermethrin, the tolerance ranges from 8 to 40 ppm for animal feed forage and hay (nongrass), and a lower tolerance of 0.05 ppm is established for “feed commodities (other than those covered by a higher tolerance as a result of use on growing crops)” [22]. The tolerance for deltamethrin ranges from 0.5 to 10 ppm for sorghum forage, field corn forage, and sweet corn forage, and a lower tolerance of 0.05 ppm is established for deltamethrin residues in or on “feed items (other than those covered by a higher tolerance as a result of use on growing crops)” [22].

Based on these findings, the concentration of PBO in all animal feed samples analyzed was below the EPA tolerance level, assuming that the residues were solely the result of contamination from storage bags. For cypermethrin exposure, if we consider the animal feeds analyzed in this study as processed and mixed feeds and apply the lower EPA tolerances of 0.05 ppm, both pig feed sample 1 and poultry feed sample 2 would be categorized as feed items of concern due to the higher cypermethrin concentrations over the tolerance level. Similarly, for deltamethrin exposure, goat feed sample 1 and sheep feed sample 1 may raise higher safety concerns due to their deltamethrin levels exceeding the tolerance of 0.05 ppm. These results suggest the need to monitor PBO, pyrethroids, and pyrethrins residue contaminations in animal feeds to ensure their safety for both animal and human health.

### 2.4. Method Application to a Toxicological Case

Toxicology case samples of a commercial horse feed product were received after horses feeding on the product displayed symptoms including diarrhea, colic, anorexia, and ataxia. An in-house screening method based on GC/MS identified the presence of PBO in two lots of the submitted feed samples. As PBO is commonly used in combination with pyrethrins and pyrethroids, the samples were further analyzed using the developed LC-MS/MS method to confirm and quantify the PBO, as well as to identify any pyrethrins and pyrethroids present.

Table 4 shows that case samples from both feed lots contained PBO with concentrations ranging from 1.04 to 2.48 mg/kg. Deltamethrin was also detected in both samples at levels of 38.4 and 90.6 µg/kg, respectively. In contrast, samples 1 to 3 of AAFCO horse feeds contained PBO concentrations ranging from 4.02 to 90.1 µg/kg, with deltamethrin ranging from negative to trace levels. The toxicology case samples had significantly higher PBO and deltamethrin levels than the AAFCO horse feeds. Furthermore, the deltamethrin concentration in the Lot #2 sample exceeded the EPA tolerance of 0.05 ppm, while the level in the Lot #1 sample was slightly lower than the tolerance. While the PBO levels were below the EPA tolerance of 10 ppm, the relatively high concentrations of 1 to 2 ppm suggest a higher potential for PBO to enhance the potency of co-existing deltamethrin, leading to an elevated risk of adverse health effects in horses. It is worth noting that although the concentrations of PBO and deltamethrin differed between the Lot #1 and Lot# 2 samples, the ratio of PBO to deltamethrin remained the same at a ratio of 27:1, suggesting the same insecticide formulation was present in both lots of samples. The difference may be attributed to variations in dosage or storage conditions.

It has been reported that exposure to pyrethrins and pyrethroids accounted for all calls of insecticide poisoning in horses at a poison control center [23], suggesting these specific insecticides may play a significant role in causing poisoning incidents among horses [24]. The general clinical signs in horses are expected to be similar to those observed in dogs, cats and other large animals, which include “salivation, vomiting, hyperexcitability, tremors, seizures, dyspnea, weakness, prostration, and death” [1,4,24]. Additionally, symptoms including diarrhea, ataxia, and anorexia have been associated with exposure to deltamethrin in humans [8,10,25].

The developed method provided valuable information regarding insecticide contaminants’ identity, concentration, and profile in the suspected feeds. This information, coupled with relevant clinical data, can aid veterinary diagnosticians and toxicologists in identifying the underlying causes of symptoms and devising effective treatment plans.

## 3. Conclusions

In this study, a simple and rapid LC-MS/MS method was developed to simultaneously determine pyrethrins, pyrethroids, and PBO in animal feeds. The method demonstrated good sensitivity, accuracy, and precision, indicating its suitability for routine analysis. The method was successfully utilized to determine the insecticide contaminations in a variety of livestock and poultry feeds and a toxicology case sample, highlighting its potential utility in animal health and food safety applications, as well as veterinary toxicology investigations. Further studies can be conducted to expand the method’s scope to include additional pyrethroids and matrices.

## 4. Materials and Methods

### 4.1. Chemicals and Reagents

Standards of cypermethrin, deltamethrin, permethrin, and pyrethrins were purchased from AccuStandard (New Haven, CT, USA). PBO, PBO-d_9_, cypermethrin-d_5_, and permethrin-d_5_ were purchased from Sigma-Aldrich (St. Louis, MO, USA). Deltamethrin-d_5_ was obtained from Cayman Chemical (Ann Arbor, MI, USA). The pyrethrins were a certified mixture with the following components: pyrethrin I—26.6%, pyrethrin II—13.16%, cinerin I—2.70%, cinerin II—1.80%, jasmolin I—1.89%, and jasmolin II—1.26%. The pyrethroid and deuterated pyrethroid standards were a mixture of isomers. Stock solutions of individual standards were prepared at a concentration of 1 mg/mL and stored at −20 °C. A working solution of the standard mixture was then prepared from individual stock solutions and further diluted to construct the calibration curve. QuEChERS (Quick, Easy, Cheap, Effective, Rugged, and Safe) extraction and dispersive salts were obtained from Agilent Technologies (Santa Clara, CA, USA). In total, 1 g of QuEChERS extraction salts contains approximately 0.615 g magnesium sulfate, 0.154 g sodium chloride, 0.154 g sodium citrate, and 0.077 g disodium citrate sesquihydrate; 1 g of QuEChERS dispersive salts contains 0.2 g PSA, 0.2 g C18, and 0.6 g magnesium sulfate. Acetonitrile and methanol (LC/MS grade) were obtained from Fisher Scientific (Waltham, MA, USA). Ammonium acetate and formic acid were obtained from Sigma-Aldrich (St. Louis, MO, USA). Ultrapure water (18.2 MΩ·cm) was obtained from a Milli-Q Integral system from Millipore (Burlington, MA, USA).

### 4.2. Sample Preparation

The animal feed samples were extracted using QuEChERS-based methods [26]. First, the feed samples were ground and milled if they were not pre-ground. Then, the ground feed weighing 0.1 g was placed in a polypropylene centrifuge tube, to which deuterated IS solutions were added. Then, 0.2 mL of ultrapure water and 0.8 mL of acetonitrile were added to the tube. The sample was vortexed for 10 min and centrifuged at 13,000× *g* for 5 min. The resulting supernatant was then added to a tube containing 0.1 g of QuEChERS extraction salts and vortexed for 1 min. After the tube was centrifuged at 13,000× *g*, 0.4 mL of the supernatant was transferred to a tube containing 0.1 g of QuEChERS dispersive salt and vortexed for another 1 min. Subsequently, 0.2 mL of the final supernatant was diluted with 0.8 mL 50/50 (*v*/*v*) methanol/water and centrifuged at 13,000× *g* to pellet down any particulates. Finally, 0.1 mL of the diluted supernatant was transferred to a glass insert in an autosampler vial for LC-MS/MS analysis. The final dilution factor was 50.

### 4.3. LC-MS/MS Instrument Analysis

LC-MS/MS analysis was conducted using an Agilent 1290 Infinity LC system coupled with an Agilent 6470A triple quadrupole mass spectrometer. The chromatographic separation was performed using a Poroshell 120 column (EC-C18, 2.1 × 50 mm, 2.7 µm) coupled to a guard column (EC-C18, 2.1 × 5 mm, 2.7 µm). The flow rate was maintained at 0.4 mL/min with the column temperature set at 40 °C. The autosampler was kept at 10 °C and the injection volume was 5 µL. The mobile phases comprised (A) 5 mM ammonium acetate in water and (B) 5 mM ammonium acetate in methanol. A gradient profile was utilized for the separation. Specifically, the initial mobile phase (B) was held at 70% for 0.5 min, followed by a gradual increase to 95% over a duration of 3 min. The mobile phase (B) was maintained at 95% for 2 min and then returned to 70%. The mass spectrometry was carried out in multiple reaction monitoring (MRM) modes. All the compounds were monitored in positive electrospray ionization (ESI^+^) mode with the following MS source conditions: dry gas temperature at 275 °C, dry gas flow at 8 L/min, nebulizer at 30 psi, sheath gas temperature at 225 °C, sheath gas flow at 10 L/min, capillary at 4000 V (positive). For each compound, two transitions of precursor/product ion were employed, one for quantification and the other for qualification (see Table 5). The data were acquired and analyzed using MassHunter software (version 10.0, Agilent Technologies, Santa Clara, CA, USA, 2018).

### 4.4. Method Validation

The developed method was validated based on the Guidelines for the Validation of Chemical Methods for the FDA Foods Program and research studies on method development and validation [27,28,29]. A range of parameters were evaluated, including linearity, accuracy, precision, matrix effects, processed sample stability, LOD, LOQ, and dilution integrity.

Briefly, the calibration curve was constructed in solutions using the ratio of the peak area of the analyte to that of the corresponding IS. The linearity of the calibration curve was evaluated using linear least-squares regression over three batches on three different days. To evaluate the accuracy and precision of the method, blank feed samples were spiked at three different concentrations and utilized as quality control (QC) samples (see Table 3). Accuracy is calculated as the percentage of measured concentration of spiked samples compared to the theoretically added concentration [30]. Intra-day and inter-day assessments were conducted by analyzing the QC samples on three separate days. To determine the impact of matrix effects on the ionization of these compounds, peak intensities of the post-spiked feed samples were compared to those of the neat standards. For assessing the stability of processed samples, QC samples of medium concentration were stored in an autosampler at 10 °C to assess the stability of processed samples. The samples were analyzed over five consecutive days.

In addition, the LOQ was established as the concentration of spiked feed samples at the lowest calibrator level with an S/N ratio of 10 or higher and an accuracy between 80 and 120%. The LOD was calculated based on an S/N ratio of 3 [28,29]. To assess dilution integrity, a QC sample with analyte concentrations greater than the highest calibrator level was prepared by spiking a blank feed sample with 1000 µg/kg of PBO, 10,000 µg/kg of pyrethroids, and 270–5610 µg/kg of pyrethrins. The fortified QC sample was diluted 50 times by thoroughly mixing 0.1 g of QC samples with 4.9 g of blank feed. The resulting diluted sample was then analyzed in five replicates to assess the accuracy and precision for 1:50 (*w*/*w*) dilution.

### 4.5. Real Feed Samples

Ground animal feeds for cattle, horses, goats, sheep, pigs, and poultry were obtained from the AAFCO (IL, USA). The toxicology samples were from submissions of 2 lots of a commercial horse feed product, which were milled and ground before analysis.

## Figures and Tables

**Table 1:** Quantitation range, LOD, LOQ, and matrix effects of target compounds in animal feeds.

Compound	Quantitation Range (µg/kg)	LOD (µg/kg)	LOQ (µg/kg)	Matrix Effects (%)
PBO	1–50	0.15	1	108
Cypermethrin	10–500	3	10	82
Deltamethrin	10–500	3	10	77
Permethrin	10–500	3	10	76
Pyrethrin I	5.61–280.5	1.68	5.61	84
Pyrethrin II	2.78–139	0.83	2.78	82
Cinerin I	2.85–28.5	0.86	2.85	88
Cinerin II	1.9–19	0.57	1.9	86
Jasmolin I	2–20	0.60	2	87
Jasmolin II	1.35–13.5	0.41	1.35	84

**Table 2:** Research studies reported for the determination of pyrethrins, pyrethroids, or PBO in animal-derived food or feed.

Compound	Analytical Technique	Sample Type	Sample Size (g or mL)	Run Time (min)	LOD (µg/kg)	LOQ (µg/kg)	Reference
Pyrethroids	GC/MS	Fish tissue	10	25	N.A.	5–10	[12]
Pyrethroids	GC-MS/MS	Beef, pork, chicken, milk, and eggs	5	26.83	N.A.	10	[14]
Pyrethroids, Organochlorine	GC/MS	Beef meat	10	~60	N.A.	13–125	[16]
Pyrethrins, Pyrethroids	LC-MS/MS	Animal fat	1	21	N.A.	50	[19]
					N.A.	10–500	
PBO, Other insecticides	LC-MS/MS	Porcine muscle, eel, flatfish, shrimp, milk, andwhole egg	2	8	0.3–0.7	0.6–1.5	[20]
PBO, Other pesticides Veterinary drugs	LC-MS/MS	Dairy cattle feed	5	21	5	15	[21]
Pyrethrins	LC-MS/MS	Various animal feed for cattle, horses, goats, sheep, pigs, and poultry	0.1	<7	0.41–1.68	1.35–5.61	This study
Pyrethroids					3	10	
PBO					0.15	1	

N.A.: not available.

**Table 3:** Accuracy and precision of the target compounds in spiked feed samples.

Compound	Spiked Level (µg/kg)	Intra-Day (%, n = 5)		Inter-Day (%, n = 15)	
		Accuracy	Precision	Accuracy	Precision
PBO	1	112	4.2	109	3.9
	20	97	1.4	97	1.2
	50	104	1.7	103	1.8
Cypermethrin	10	92	7.2	94	9.1
	200	86	4.6	87	3.9
	500	84	2.9	86	2.8
Deltamethrin	10	91	7.6	99	8.0
	200	103	3.8	102	2.8
	500	110	2.2	108	2.2
Permethrin	10	115	4.3	109	8.2
	200	106	7.6	103	6.3
	500	114	7.4	109	6.8
Pyrethrin I	5.61	111	7.5	107	6.3
	112.2	111	4.0	110	3.3
	280.5	114	4.4	112	3.5
Pyrethrin II	2.78	114	5.3	111	4.6
	55.6	112	4.1	111	3.5
	139	114	3.5	113	3.2
Cinerin I	2.85	111	6.2	110	4.7
	11.4	112	5.1	109	4.2
	28.5	113	4.1	111	3.0
Cinerin II	1.9	107	9.0	106	7.0
	7.6	109	5.1	108	4.6
	19	113	4.2	111	3.6
Jasmolin I	2	112	4.6	111	4.2
	8	110	3.8	109	3.2
	20	112	4.7	111	3.8
Jasmolin II	1.35	113	4.5	110	4.3
	5.4	111	4.9	110	4.4
	13.5	113	4.0	111	3.6

**Table 4:** Target insecticide concentrations in a variety of livestock and poultry feeds and the toxicology case samples (n = 3).

Sample	PBO	Cypermethrin	Deltamethrin	Permethrin	Pyrethrins
Cattle feed (µg/kg)					
Sample 1	3.59	ND	Traces	ND	ND
Sample 2	20.1	ND	Traces	ND	ND
Sample 3	186.5	ND	10.3	ND	ND
Sample 4	18.3	ND	Traces	ND	ND
Sample 5	6.2	ND	Traces	ND	ND
Sample 6	1.17	ND	ND	ND	ND
Horse feed (µg/kg)					
Sample 1	4.02	ND	ND	ND	ND
Sample 2	13	ND	Traces	ND	ND
Sample 3	90.1	ND	Traces	ND	ND
Goat feed (µg/kg)					
Sample 1	895	ND	89.6	ND	ND
Sample 2	7.87	ND	ND	ND	ND
Sheep feed (µg/kg)					
Sample 1	83.4	ND	55	ND	ND
Sample 2	14.7	ND	10.4	ND	ND
Sample 3	7.6	ND	ND	ND	ND
Sample 4	4.95	ND	ND	ND	ND
Pig feed (µg/kg)					
Sample 1	6.04	77.2	ND	ND	ND
Sample 2	1.18	ND	ND	ND	ND
Sample 3	32.2	Traces	ND	ND	ND
Sample 4	11.6	Traces	ND	ND	ND
Poultry feed (µg/kg)					
Sample 1	4.75	ND	ND	ND	ND
Sample 2	6.28	93.5	Traces	ND	ND
Sample 3	2.46	ND	ND	ND	ND
Toxicology case (µg/kg)					
Lot #1 Sample	1040	ND	38.4	ND	ND
Lot #2 Sample	2480	ND	90.6	ND	ND

ND: not detected (<LOD). Traces: values between LOD and LOQ.

**Table 5:** Precursor/product ions and internal standards used for the target compounds.

Compound	Precursor Ion (*m*/*z*)	Product Ion for Quantification (*m*/*z*)	Product Ion for Qualification (*m*/*z*)	Collision Energy (eV) *	Internal Standard
PBO	356.2	177.0	119.0	9, 41	PBO-d_9_
Cypermethrin	433.1	190.9	416.1	13, 5	Cypermethrin-d_5_
Deltamethrin	523.0	280.9	506.0	13, 5	Deltamethrin-d_5_
Permethrin	408.1	183.0	355.1	21, 5	Permethrin-d_5_
Pyrethrin I	329.2	160.9	143.0	5, 17	Cypermethrin-d_5_
Pyrethrin II	373.2	161.1	133.1	5, 25	Cypermethrin-d_5_
Cinerin I	317.2	149.1	107.0	5, 21	Cypermethrin-d_5_
Cinerin II	361.2	149.0	107.0	5, 25	Cypermethrin-d_5_
Jasmolin I	331.2	163.1	107.0	5, 21	Cypermethrin-d_5_
Jasmolin II	375.2	163.1	107.0	5, 33	Cypermethrin-d_5_

PBO: piperonyl butoxide. * The first value for quantification ion transition and the second one for qualification ion transition.

## Data Availability

The data presented in this study are available in this article.
